# Dietary selenium augments sarcoplasmic calcium release and mechanical performance in mice

**DOI:** 10.1186/s12986-016-0134-6

**Published:** 2016-11-03

**Authors:** Dóra Bodnár, Olga Ruzsnavszky, Tamás Oláh, Beatrix Dienes, Ildikó Balatoni, Éva Ungvári, Ilona Benkő, Beáta Babka, József Prokisch, László Csernoch, Péter Szentesi

**Affiliations:** 1 Department of Physiology, Faculty of Medicine, University of Debrecen, P.O. Box 400, H-4002 Debrecen, Hungary; 2 Department of Pharmacology and Pharmacotherapy, Faculty of Medicine, University of Debrecen, Debrecen, Hungary; 3 Institute of Animal Science, Biotechnology and Nature, Faculty of the Agricultural and Food Sciences and Environmental Management, University of Debrecen, Debrecen, Hungary

**Keywords:** Selenium, Skeletal muscle, Force, Intracellular calcium concentration, Selenoprotein

## Abstract

**Background:**

As an essential trace element selenium plays a significant role in many physiological functions of the organs. It is found within muscles as selenocystein in selenoprotein N, which is involved in redox-modulated calcium homeostasis and in protection against oxidative stress.

**Methods:**

The effects of two different selenium compounds (selenate and NanoSe in 0.5 and 5 ppm concentration for two weeks) on muscle properties of mice were examined by measuring *in vivo* muscle performance, *in vitro* force in *soleus* (SOL) and *extensor digitorum longus* (EDL) muscles and changes in intracellular Ca^2+^ concentration in single fibers from *flexor digitorum brevis* (FDB) muscle.. Western-blot analysis on muscle lysates of EDL and SOL were used to measure the selenoprotein N expression. Control mice received 0.3 ppm Se.

**Results:**

While the grip force did not change, 5 ppm selenium diets significantly increased the speed of voluntary running and the daily distance covered. Both forms of selenium increased significantly the amplitude of single twitches in EDL and SOL muscle in a concentration dependent manner. Selenate increased fatigue resistance in SOL. The amplitude of the calcium transients evoked by KCl depolarization increased significantly from the control of 343 ± 44 nM to 671 ± 51 nM in the presence of 0.5 ppm selenate in FDB fibers. In parallel, the rate of calcium release during short depolarizations increased significantly from 28.4 ± 2.2 to 45.5 ± 3.8 and 52.1 ± 1.9 μM/ms in the presence of 0.5 ppm NanoSe and selenate, respectively. In 0.5 ppm concentration both selenium compounds increased significantly the selenoprotein N expression only in EDL muscle.

**Conclusions:**

Selenium supplementation augments calcium release from the sarcoplasmic reticulum thus improves skeletal muscle performance. These effects are accompanied by the increased selenoprotein N expression in the muscles which could result in increased oxidative stress tolerance in case of long lasting contraction.

**Electronic supplementary material:**

The online version of this article (doi:10.1186/s12986-016-0134-6) contains supplementary material, which is available to authorized users.

## Background

Significant proportion of locomotor diseases is caused by muscle weakness or wasting, but the biological mechanisms involved in these processes are not completely understood. One potential cause could be the accumulation of nuclear and mitochondrial DNA damage and increases in oxidative stress which leads to the loss of muscle fibers and, thus, decreased functionality of skeletal muscle [1]. Selenium as a trace element with antioxidant properties plays an important role in muscle function [2], and associated enzymes as glutathione reductase and peroxidase protect muscle fibers from reactive oxygen species [3].

Selenium is a trace mineral needed in small amounts each day. It is well known for its antioxidant properties which are responsible for its antiviral and anticancer abilities (for review see [4]), but it is also important for normal muscle function. Skeletal muscle disorders manifesting in muscle pain, fatigue, proximal weakness, and serum creatine kinase elevation have been reported in patients with selenium deficiency [5]. Recent experiments on transgenic mice lacking the antioxidant enzyme superoxide dismutase have shown an acceleration of sarcopenia due to neuromuscular degeneration following mitochondrial dysfunction. This finding indicates that a decline in the ability to cope with an increase in oxidative stress may be, at least in rodents, an important cause of sarcopenia [6]. In ruminant animals, selenium deficiency causes white muscle disease, the symptoms of which are muscle weakness and degeneration of cardiac and skeletal muscle [7]. Similar human myopathy has also been described [8] and severe selenium deficiency is associated with skeletal muscle problems [5].

The recommended daily dietary allowance (RDA) for selenium according to the European Union is 20–75 μg for adults and 10–25 μg for children [9]. Unfortunately, the selenium intake is below the recommended amount in Europe at present [10]. Therefore, there are extensive actions to initiate selenium supplementation in the population, however, because its toxicity in high concentration the results are diverse and sometime controversial.

The supplementation of vitamins and elements is very important and one way to do this is the application of nanoparticles. They have distinctive properties and extensive application in the healthy diet. Among the various types of nanoparticles, selenium has good absorption capacity, lower toxicity, high bioavailability and interaction with proteins [11]. In the past few years a new type of nano-selenium (NanoSe) was developed as nano sized elemental selenium particles (between 100 and 500 nm; [12]). It has lower toxicity and better physiological effects than inorganic mineral forms of selenium. Recent studies proved that the toxicity of Se forms decreased in the following order: selenate > selenite > NanoSe > LactoMicroSel® [13, 14]. NanoSe is produced by lactic acid bacteria (LactoMicroSel® is the same, but made of milk producing dried yoghurt powder). While selenate is absorbed as is, NanoSe is transformed into selenite *in vivo*.

The biological functions of Se are associated with selenoproteins, a family of proteins which contain this micronutrient in the form of selenocysteine. The functions of several selenoproteins have been described, but most of them have no known functions. Its conservation among species and their preserved production pathway highlight the importance of these proteins. Most of those selenoproteins whose function is identified are catalytically active in redox processes. One of them is glutathione peroxidase (GPx) which plays a role in the catalytic decomposition of H_2_O_2_ into H_2_O and oxygen. In mammals, there are several selenocysteine-containing GPx and they modify the oxidation of reduced glutathione (GSH) which is present in millimolar concentrations in muscle fibers. Nevertheless, the only selenoprotein implicated so far in a human genetic disorder associated with skeletal muscles is selenoprotein N (SelN), encoded by the SEPN1 gene [15] which manifests in muscle dystrophy. It was shown that SelN is involved in redox-modulated calcium homeostasis in skeletal muscle [16] so it could alter the contraction.

Shortening of muscle is a multi-step process called excitation contraction (EC) coupling. The trigger action potential traveling along the sarcolemma and the transverse-(T-) tubule membranes activates the calcium release from the intracellular store (sarcoplasmic reticulum, SR) via Ca^2+^ release channels (ryanodine receptor, RyR; [17]). The voltage sensor/L-type calcium channel in the T-tubular membrane (dihydropyridine receptor, DHPR; [18]) activates RyR via a direct conformational coupling. The released calcium ions bind to the regulatory sites on troponin-C and initiate muscle contraction. During relaxation calcium is moved back into the SR via the sarcoplasmic calcium pump (SERCA). Since both RyR and SERCA activity are altered by oxidation, the antioxidant selenium could have an impact on EC-coupling by modifying intracellular calcium homeostasis.

The specific aim of the present project was to investigate the steps of EC coupling on selenium fed mice and to understand how its modification leads to the observed changes in muscle performance.

## Methods

### Chemicals and selenium sources

Chemicals, unless otherwise stated, were purchased from Sigma (St. Louis, MO, USA) and were of analytical grade.

Inorganic mineral form of selenate (sodium selenate, Na_2_SeO_4_) and selenite (sodium hydrogen selenite, NaHSeO_3_) were purchased from CEDA Chemicals (Duelmen, Germany). For producing nano-sized (100 to 500 nm diameter) elemental selenium (NanoSe) we used the inorganic selenite dissolved in 5 % MRS Broth bacteria culture media. The selenite containing media was inoculated with *Lactobacillus casei* probiotic yoghurt bacteria, freshly produced from freeze-dried bacteria (three times re-inoculation). The final concentration of selenium was 200 mg/L. The prepared solution was fermented at 37 °C in a shaking incubator for 48 h. After the fermentation the red color of the elemental selenium nano-sized balls produced by the bacteria can be seen (see Additional file 1: Figure S1). To extract the pure NanoSe particles we centrifuged MRS, digested the bacteria pellet with cc. hydrochloric acid, and washed as well as filtered the cell debris out. The final NanoSe is a sol (a colloidal suspension of solid particles with diameter below 500 nm).

### Animal care

Animal experiments conformed to the guidelines of the European Community (86/609/EEC). The experimental protocol was approved by the institutional Animal Care Committee of the University of Debrecen (4/2011/DE MAB). The 25-week-old BDF1 male mice weighing an average of 27 g were housed in plastic cages with mesh covers, and fed with pelleted mouse chow and water *ad libitum*. Room illumination was an automated cycle of 12 h light and 12 h dark, and room temperature was maintained within the range 22–25 °C.

The animal model used was described previously [13]. In brief, inorganic sodium selenate and NanoSe was administered for 14 days at concentrations of 0.5, and 5 ppm (particle per million) Se, equivalent to 0.5, and 5 mg Se/kg food, corresponding to an estimated 4, and 40 μg/kg body weight/day Se uptake, respectively. The length of feeding and the applied concentrations were chosen to minimize toxicity of selenium. Forming lab diet we minced the pelleted mouse chow and mixed carefully with the appropriate amount of distillated water dissolved sodium selenate, as well as homogenized NanoSe sol. After mixing we granulated and dried the selenium containing chow again. The concentration of the NanoSe sol and selenate in the mixed chows was verified with atomic fluorescence spectrometer after nitric acid destruction. The control lab diet [19] was obtained from Charles River (Wilmington, MA, USA) and it contains sodium selenite. Its 0.3 ppm Se content was subtracted from the administered Se doses. The control group received no additional Se besides that of the basal diet. The number of animals in the groups is presented in Table 1. Bodyweights of the animals were measured daily as well as their daily food intake.

**Table 1:** Body weight changes, muscle/body weights and cross sectional areas

		Control (10)	Selenate		NanoSe	
			0.5 ppm (6)	5 ppm (8)	0.5 ppm (6)	5 ppm (4)
Body weight change (g)		0.6 ± 0.2	2.3 ± 0.2*	3.6 ± 0.3*	2.5 ± 0.3*	1.3 ± 0.3*
Muscle/body weight (%)	EDL	0.038 ± 0.003 (14)	0.050 ± 0.01^#^ (11)	0.045 ± 0.001 (10)	0.051 ± 0.001^#^ (10)	0.049 ± 0.001^#^ (7)
	SOL	0.038 ± 0.001 (10)	0.056 ± 0.002^#^ (12)	0.046 ± 0.002^#^ (15)	0.056 ± 0.001^#^ (10)	0.049 ± 0.004^#^ (7)
Cross sectional area (mm^2^)	EDL	1.75 ± 0.13	1.95 ± 0.16	1.87 ± 0.11	1.84 ± 0.13	1.58 ± 0.15
	SOL	1.49 ± 0.13	1.54 ± 0.79	1.25 ± 0.06	1.68 ± 0.20	1.24 ± 0.11

*denotes significant increase (*P* < 0.05) compared to the starting body weight

^#^denotes significant difference (*P* < 0.05) compared to control

Numbers in parenthesis denote the number of animals or muscles

Animals were anaesthetized and sacrificed following a protocol approved by the Animal Care Committee of the University of Debrecen. After pentobarbital anesthesia (27 mg/kg), the *m. flexor digitorum brevis* (FDB), the *m. extensor digitorum longus* (EDL) and *m. soleus* (SOL) from the hind limb were dissected.

#### In vivo experiments

##### Voluntary activity wheel measurement

Mice from different groups of treatment were singly housed in a cage with a mouse running wheel (Campden Instruments Ltd., Loughborough, UK). Wheels were interfaced to a computer and revolutions were recorded in 20 min intervals, continuously for 10 days. The average and the maximal speed, the distance and the duration of running was calculated for the individual mice and then averaged by treatments.

##### Forepaw grip test

The force of forepaw was measured as described earlier [20]. Briefly, when the animals reliably grasped the bar of the grip test meter, they were then gently pulled away from the device. The maximal force before the animal released the bar were digitized at 2 kHz and stored by an online connected computer.

#### In vitro experiments

##### Measurement of muscle force

Muscle contractions were measured as described in our previous reports [21]. In brief, fast and slow twitch muscles, EDL and SOL, were removed and placed horizontally in an experimental chamber continuously super fused (10 ml/min) with Krebs’ solution (containing in mM: NaCl 135, KCl 5, CaCl_2_ 2.5, MgSO_4_ 1, Hepes 10, glucose 10, NaHCO_3_ 10; pH 7.2; room temperature) equilibrated with 95 % O_2_ plus 5 % CO_2_. One end of the muscle was attached to a rod while the other to a capacitive mechano-electric force transducer. Two platinum electrodes placed adjacent to the muscle were used to deliver short, supramaximal pulses of 2 ms in duration to elicit single twitches. Force responses were digitized at 2 kHz using Digidata 1200 A/D card and stored with Axotape software (Axon Instruments, Foster City, CA, USA). Muscles were then stretched by adjusting the position of the transducer to a length that produced the maximal force response and allowed to equilibrate for 60 min.

Single pulses at 0.5 Hz were used to elicit single twitches. At least 10 twitches were measured under these conditions from every muscle. The individual force transients within such a train varied by less than 3 % in amplitude, thus the mean of the amplitude of all transients was used to characterize the given muscle. To elicit a tetanus, single pulses were applied with a frequency of 200 Hz for 200 ms (EDL) or 100 Hz for 500 ms (SOL). Duration of individual twitches and tetani were determined by calculating the time between the onset of the transient and the relaxation to 90 % of maximal force.

##### Isolation of single skeletal muscle fibers

All calcium concentration measurements were carried out on skeletal muscle fibers from the FDB muscle of the mouse. Calcium free Tyrode’s solution (145 mM NaCl, 2.7 mM KCl, 1 mM MgCl_2_, and 11.8 mM Hepes, pH 7.4) was used during the dissection of the muscle. Single muscle fibers from FDB were enzymatically dissociated in minimal essential media containing 0.2 % Type I collagenase (Sigma) at 37 °C for 65 min [22, 23]. To release single fibers, the FDB muscles were triturated gently in normal Tyrode’s solution (1.8 mM CaCl_2_). The fibers were then placed in culture dishes and stored at 4 °C in refrigerator until use.

##### Whole cell intracellular Ca^2+^ concentration measurement

Intracellular Ca^2+^ concentration ([Ca^2+^]_i_) was monitored using Fura-2 in acetoxymethyl form as described previously [22, 24]. Briefly, isolated FDB fibers were mounted on a laminin-coated cover slip and loaded with 5 μM Fura-2 AM for 60 min. Fibers were then equilibrated in Tyrode’s solution for 30 min at room temperature. Cover slips with Fura-2 loaded fibers were placed on the stage of an inverted fluorescence microscope (Diapoth, Nikon, Tokyo, Japan). The excitation wavelength was altered between 340 and 380 nm by a microcomputer-controlled dual-wavelength monochromator (Deltascan, Photon Technology International, New Brunswick, NJ), whereas the emission was monitored at 510 nm using a photomultiplier at 10 Hz acquisition rate of the ratios at 22 °C. Ca^2+^transients were evoked by KCl depolarization. Fibers were permanently washed with Tyrode’s solution using a background perfusion system, whilst the depolarizing solution (120 mM NaCl was replaced by 120 mM KCl) was applied through a local perfusion system, which was positioned in close proximity to the measured fiber. Intracellular calcium concentration was calculated from the ratio of measured fluorescence intensities at 340 and 380 nm using an *in vivo* calibration as described in our earlier report [22].

##### Confocal intracellular Ca^2+^ concentration measurement

Changes in [Ca^2+^]_i_ were also monitored using Rhod-2 AM as described previously [20]. Briefly, after dissociation, FDB fibers were placed into culture dish with Tyrode’s solution. Depolarization-evoked calcium transients were measured at 22 °C using a confocal laser scanning microscope system (Zeiss 5 Live, Oberkochen, Germany) after loading the fibers with 20 μM Rhod-2 AM for 15 min at room temperature. Individual action potentials were evoked by applying supra-threshold 2 ms long square pulses (S88 Stimulator, Grass Technologies, Warwick, RI, USA) through a pair of platinum electrodes placed close to the fiber. Line-scan images (512 pixels/line) were used to monitor the fluorescence intensity changes at 1 ms/line and using a 40x water immersion objective. Rhod-2 was excited with a HeNe ion laser at 543 nm, emission was detected with a 550 nm long pass filter. To obtain the time-course of Rhod-2 fluorescence change (F_rhod_), corresponding data points (usually 10–15) in the line-scan images were averaged in the spatial domain. Resting fluorescence was determined as the average fluorescence before the depolarization. Changes in [Ca^2+^]_i_ were then calculated using the formula

1 $$ {\left[{\mathrm{Ca}}^{2+}\right]}_{\mathrm{i}} = {\mathrm{Kd}}_{\mathrm{rhod}} \bullet \left(1/{\mathrm{k}}_{\mathrm{off},\ \mathrm{rhod}} \bullet {\mathrm{dF}}_{\mathrm{rhod}}/\mathrm{d}\mathrm{t} + {\mathrm{F}}_{\mathrm{rhod}}\hbox{--}\ {\mathrm{F}}_{\mathrm{rhod}, \min}\right)\ /\ \left({\mathrm{F}}_{\mathrm{rhod}, \max}\hbox{--}\ {\mathrm{F}}_{\mathrm{rhod}}\right) $$

where F_rhod,max_ and F_rhod,min_ were determined in our laboratory, while other parameters (Kd_rhod_ = k_off,rhod_ / k_on,rhod_ is the dissociation constant, k_off,rhod_ and k_on,rhod_ the backward and forward rate constant for the calcium-dye reaction, respectively) for Rhod-2 were taken from Escobar and co-workers [25].

##### Calculation of calcium release from the SR

The Ca^2+^ release flux (FL) was defined as the first time derivative of the sum of the Ca^2+^ in the myoplasmic space and that transported back into the SR, as described earlier:

2 $$ \mathrm{F}\mathrm{L} = \mathrm{d}\left({\mathrm{Ca}}_{\mathrm{total}} + {\mathrm{Ca}}_{\mathrm{transp}}\right)\ /\ \mathrm{d}\mathrm{t}, $$

where Ca_total_ is the total Ca^2+^ in the myoplasm, while Ca_transp_ is the amount of Ca^2+^ transported by the Ca^2+^ pumps [15]. Ca_total_ was estimated as the sum of free Ca^2+^ and the amount of Ca^2+^ bound to intracellular binding sites.

In Eq. 2. Ca_transp_ was considered proportional to the relative saturation of the pumps. Removal parameters were taken from the literature [26] and used to calculate the binding of Ca^2+^ to intracellular binding sites as described in our earlier reports [27].

##### Preparation of cell extracts

Freshly prepared EDL and soleus muscles were mechanically homogenized in buffer (20 mM Tris–Cl, 5 mM EGTA, 1 mM 4-(2-aminoethyl) benzenesulphonyl fluoride, 20 μM leupeptin, pH 7.4; all from Sigma) and disrupted by sonication on ice. Protein content of the samples was measured by a modified bicinchoninic acid (BCA) protein assay (Pierce, Rockford, IL, USA) using BSA as a standard.

##### Western blot analysis

For Western blot analysis (SDS–PAGE) total muscle lysates of EDL and SOL were prepared by adding 1/5 volume of fivefold concentrated electrophoresis sample buffer (310 mM Trise-HCl pH 6.8, 10 % SDS, 50 % glycerol, 100 mM DTT, 0.01 % bromophenol blue) to muscle lysates and boiled for 10 min at 80 ͦC. 30 μg of protein was separated by 7.5 % SDS–PAGE gel for immunological detection of selenoprotein N. Proteins were transferred electrophoretically to nitrocellulose membranes. After blocking in 5 % non-fat dry milk in PBS, membranes were incubated with primary antibodies overnight at 4 °C as follows: rabbit polyclonal anti-selenoprotein N antibody raised against amino acids 293–452 mapping within an internal region of selenoprotein N of human origin in 1:200, (Santa Cruz Biotechnology, INC, Santa Cruz, CA, USA); and rabbit polyclonal anti-actin antibody in 1:500 (Santa Cruz Biotechnology, Inc., Santa Cruz, CA, USA). After washing for 30 min in PBST, membranes were incubated with the HRP-conjugated secondary antibody (Bio-Rad, Hercules, CA, USA) in 1:1000 dilution in PBS containing 5 % non-fat dry milk for 1 h. Signals were detected by enhanced chemiluminescence reaction (Millipore, Billerica, MA, USA) according to the instructions of the manufacturer.

##### Statistical analysis

Pooled data were expressed as mean ± standard error of the mean (SEM). The differences between control and animals on increased *selenium diet* were assessed using one way analysis of variance (ANOVA) and all pair wise multiple comparison procedures (Student-Newman-Keuls Method) with Prism (GraphPad Software, San Diego, CA, USA). F-test was used to test the significance and a *P* value of less than 0.05 was considered statistically significant.

## Results

### In vivo experiments

Mice on selenium supplemented diet gained weight during the two weeks of Se-feeding while control animals remained almost the same weight (Table 1). To check the *in vivo* muscle performance of the animals, 4 mice were used from all groups (control, 0.5 and 5 ppm selenate and NanoSe) in grip tests. Mice from selenium-treated groups did not perform significantly better than the control animals in these tests. That is, the maximal force after normalization to body weight remained the same after 14 days on selenium diet (Table 2).

**Table 2:** Maximal grip force (mN) at the beginning and at the end of experiment

	Control (4)	Selenate		NanoSe	
		0.5 ppm (4)	5 ppm (4)	0.5 ppm (4)	5 ppm (4)
Beginning^a^	507 ± 19	534 ± 9	455 ± 9	493 ± 17	571 ± 8
End^a^	504 ± 16	509 ± 7	474 ± 14	502 ± 11	553 ± 9

Numbers in parenthesis denote the number of animals. ^a^Average of values measured on the first and last day of 14 days special diet

**Fig. 1:**
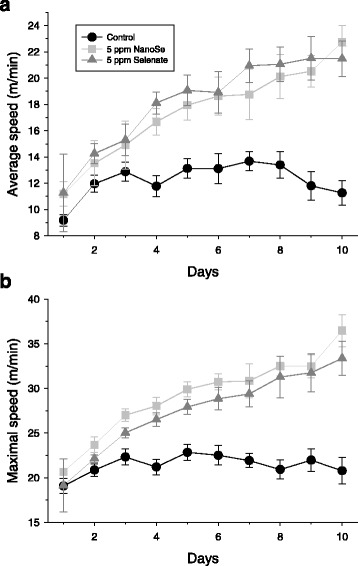
Speed of voluntary running increased after selenium treatment. Average (**a**) and maximal (**b**) speed of animals during voluntary running. After 3 days the speeds stabilized in the control group due to their accommodation to the experimental conditions

The voluntary wheel experiments showed a positive effect of selenium treatment on the performance of animals, but only in the higher (5 ppm) concentration. After 2–3 days accommodation in the special cage the average and maximal speed of running stabilized on a given level in the control group, while they increased continuously in the 5 ppm selenium treated mice (Fig. 1). The total distance covered was significantly higher in the 5 ppm selenium feed groups (Table 3). In addition, they spent more time in the wheel than control mice did at the end of 5 ppm selenium supplementation. Overall, this experiment clearly indicated, that there is a positive effect on voluntary muscle performance of selenium treatment in mice. To clarify the underlying mechanisms the steps in EC coupling were investigated in detail.

**Table 3:** Parameters of voluntary running at the beginning and at the end of experiment

		Control (8)	Selenate		NanoSe	
			0.5 ppm (8)	5 ppm (8)	0.5 ppm (8)	5 ppm (8)
Distance (m/day)	Beginning^a^	5313 ± 406	5280 ± 548	4959 ± 226	5343 ± 699	4994 ± 301
	End^a^	4592 ± 354	4910 ± 1056	6642 ± 168^##,^**	5178 ± 612	7820 ± 851^#,^*
Duration (min/day)	Beginning^a^	334 ± 16	306 ± 17	315 ± 10	339 ± 17	335 ± 16
	End^a^	253 ± 10**	264 ± 43	302 ± 4^##,^*	268 ± 26*	333 ± 17^##^
Average speed (m/min)	Beginning^a^	11.3 ± 1.1	12.5 ± 0.8	13.6 ± 1.2	11.7 ± 0.5	13.2 ± 1.1
	End^a^	12.0 ± 0.5	13.9 ± 0.7^#^	21.4 ± 0.1^###,^**	14.7 ± 0.3^##,^**	21.1 ± 0.8^###,^**
Maximal speed (m/min)	Beginning^a^	20.8 ± 0.9	23.2 ± 0.9	22.1 ± 1.7	20.5 ± 1.6	23.8 ± 1.8
	End^a^	21.2 ± 0.4	23.3 ± 1.3	32.1 ± 0.6^###,^**	23.2 ± 1.1	33.8 ± 1.3^###,^**

^#^, ^##^ and ^###^ denotes significant difference (*P* < 0.05, 0.01 and 0.001) compared to control

* and ** denotes significant difference (*P* < 0.05 and 0.01) compared to beginning

Numbers in parenthesis denote the number of animals

^a^Average of values measured on the first three and last three day of 10 days special diet

### In vitro experiments

#### Force measurement

**Fig. 2:**
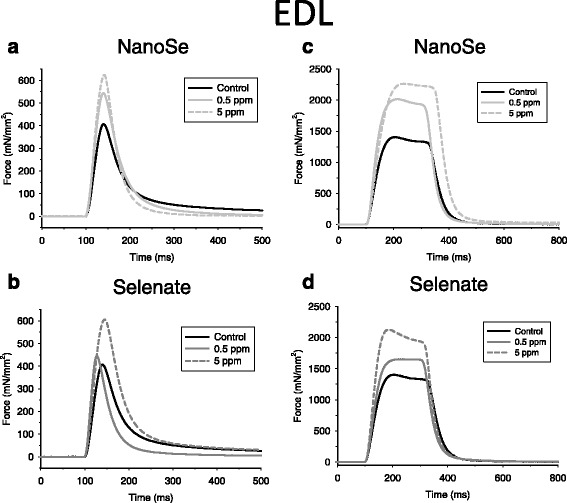
Isometric force was higher in EDL muscle of selenium treated mice. Representative twitch force (**a**, **b**) and tetanus (**c**, **d**) at two different NanoSe (**a**, **c**) and selenate (**b**, **d**) concentrations on EDL muscle at room temperature (24 °C). The force was normalized to the cross section area of the muscle

**Fig. 3:**
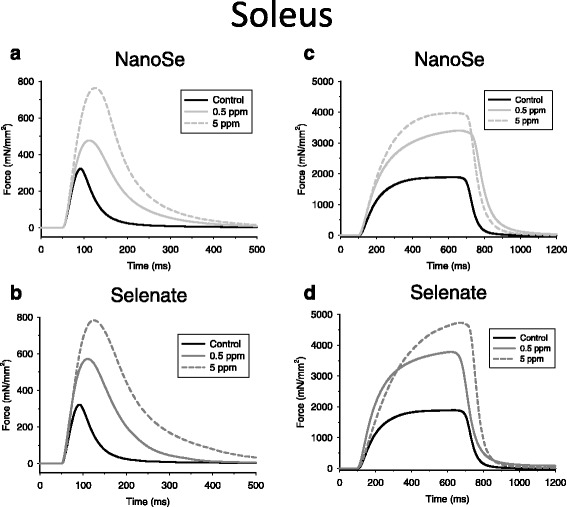
Isometric force of SOL muscle was higher in selenium treated mice. Representative twitch force (**a**, **b**) and tetanus (**c**, **d**) at two different NanoSe (**a**, **c**) and selenate (**b**, **d**) concentrations on SOL muscle at room temperature (24 °C)

In order to determine whether administration of selenium might alter muscle functions directly, the effects of selenium supplementation on *in vitro* muscle strength were investigated. A significant difference was found in the mean amplitude of the single twitches in both EDL (Fig. 2a,b) and in SOL (Fig. 3a,b) between selenium supplemented and the control animals (Table 4). Similarly, significant difference was found in tetani (Figs. 2c,d and 3c,d). In lower concentration of selenium (0.5 ppm) the average amplitude of twitches and tetani was significantly higher than in control conditions only in SOL. There were no differences in the twitch/tetanus ratio between the selenium supplemented and the control muscles (data not shown). There was no difference in the time-to-peak values except in SOL of the 0.5 ppm selenate treated animals, where it was significantly higher than in control mice. Furthermore, selenate increased fatigue resistance in SOL after 150 tetani (see Additional file 1: Figure S2). In addition, we observed a significant increase in all but one relative EDL and SOL muscle weight (Table 1).

**Table 4:** Average parameters of twitch and tetanus of EDL and soleus muscles

	Contraction type	Control		Selenate				NanoSe			
				0.5 ppm		5 ppm		0.5 ppm		5 ppm	
		EDL	SOL	EDL	SOL	EDL	SOL	EDL	SOL	EDL	SOL
Peak (mN/mm^2^)	Single twitch	0.72 ± 0.09	0.52 ± 0.10	0.68 ± 0.11	0.93 ± 0.13*	0.98 ± 0.08*	1.06 ± 0.15*	0.82 ± 0.13	1.04 ± 0.20*	0.96 ± 0.08*	1.26 ± 0.12*
	Tetanus	2.55 ± 0.39	2.72 ± 0.41	2.81 ± 0.09	5.72 ± 0.73*	3.81 ± 0.46*	6.46 ± 0.90*	3.29 ± 0.44	5.36 ± 1.31*	3.97 ± 0.56*	7.12 ± 0.37*
Time to peak (ms)	Single twitch	26.7 ± 2.0	63.5 ± 4.1	28.9 ± 1.6	68.4 ± 3.2	30.0 ± 1.3	68.2 ± 2.7	29.4 ± 1.5	65.7 ± 3.4	27.9 ± 0.73	69.4 ± 7.5
	Tetanus	142.2 ± 15.6	475.2 ± 21.2	140.1 ± 25.6	530.5 ± 4.2*	144.1 ± 10.6	458.4 ± 45.2	128.1 ± 14.4	475.5 ± 49.9	147.2 ± 12.6	493.3 ± 27.8
Fatigue after 150 tetani (%)		31.8 ± 5.4	29.5 ± 2.7	38.8 ± 10.5	43.3 ± 3.9	41.1 ± 3.6	47.5 ± 5.6	42.9 ± 3.2	45.7 ± 6.2	43.9 ± 6.3	49.7 ± 8.0
Number of muscles tested	Single twitch	14	10	11	12	14	15	10	10	7	7
	Tetanus	14	15	5	8	14	11	8	8	5	6

*denotes significant difference (*P* < 0.05) compared to control. The amplitude of the 150^th^ tetani were normalized to the first tetanus

#### Intracellular Ca^2+^ concentration changes after selenium treatment

**Fig. 4:**
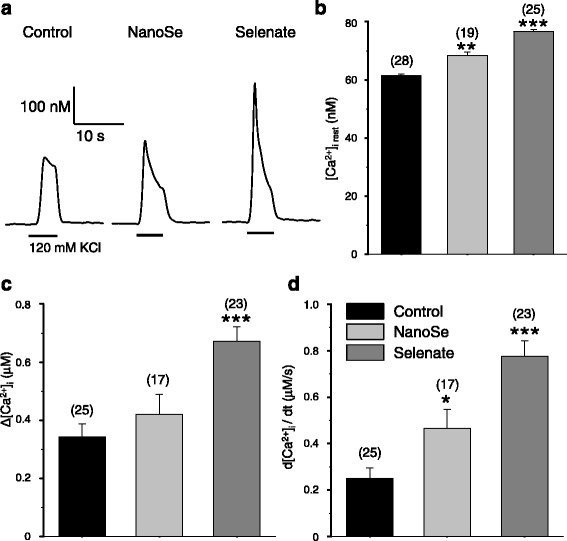
Selenium treatment augmented KCl depolarization evoked Ca^2+^ transients and resting [Ca^2+^]_i_ in FDB fibers. Depolarization evoked changes in intracellular calcium concentration in FDB muscle (**a**) of control (left), 0.5 ppm NanoSe (middle) and 0.5 ppm selenate (right) feed mice. Average values of resting [Ca^2+^]_i_ (**b**), change in [Ca^2+^]_i_ (∆[Ca^2+^]_i_) (**c**) and maximal rate of rise of the transients (**d**). Numbers in parenthesis give the number of fibers investigated. * and *** denote significant difference from control at levels of *P* < 0.05 and *P* < 0.001, respectively

The effects of the selenium treatments on the calcium homeostasis were examined in isolated, single FDB skeletal muscle fibers. Figure 4a presents representative calcium transients evoked by depolarization using 120 mM KCl in the presence of normal (1.8 mM) extracellular calcium concentration. Selenium already in 0.5 ppm concentration increased the amplitude of calcium transients. Figure 4b plots pooled data to confirm that the selenium treatment had a little, but statistically significant increasing effects on the resting [Ca^2+^]_i_ suggesting that the calcium leak from and the extrusion into the extracellular environment could be affected under these conditions. On the other hand, depolarization-evoked calcium transients were notably higher in selenium treated than in control fibers (Fig. 4c). Selenate almost doubled the amplitude of the calcium transients. The same concentration of NanoSe also increased it slightly, albeit this increase was not as pronounced. Further examination of the transients proved that the rate of calcium release from the SR was significantly higher after both kinds of selenium treatments (Fig. 4d).

#### Action potential-evoked calcium transients in FDB fibers

**Fig. 5:**
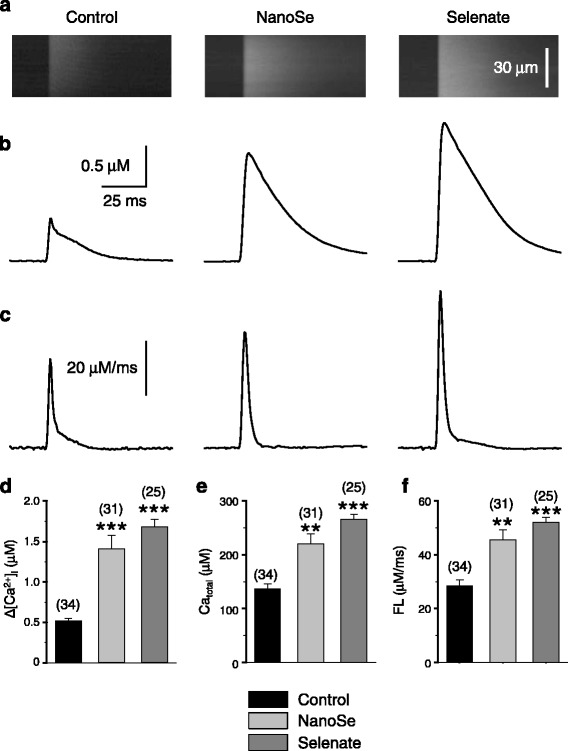
Depolarization evoked ∆[Ca^2+^]_i_ and release flux were higher in FDB fibers of selenium treated mice. Line-scan images of fluorescence intensity changes evoked by single depolarization (**a**) in FDB of control (left), 0.5 ppm NanoSe (middle) and 0.5 ppm selenate (right) feed mice. Intracellular calcium concentration changes (**b**) calculated from the Ca^2+^ transient in panel (**a**). The corresponding calcium release flux through the RyR (**c**) calculated from the curves in panel (**b**). The averages of the change in intracellular calcium concentration (**d**), the total calcium released into the myoplasm (**e**), and the corresponding calcium release flux (**f**). ** and *** show significant difference from control at levels of *P* < 0.01 and *P* < 0.001, respectively. Numbers in parenthesis give the number of fibers investigated. Measurement conditions: 8000x50 pixel, 0.5 μs/line, 0.66 μm/pixel at room temperature (24 °C)

Field stimulation-induced global calcium transients were also measured on FDB fibers. These calcium transients were visualized using laser scanning confocal microscopy in the line-scan mode. Figure 5 displays representative fluorescence intensity changes during single pulses (panel A) from control (left), 0.5 ppm selenate (middle) and NanoSe (right) treated mice. The spatial profile of the transients was homogenous in all groups showing a normal propagation of the depolarization in the T-tubular system.

To analyze the calcium transients, the line-scans were normalized to background fluorescence and 10–15 points of the images in the spatial domain were averaged and transformed to calcium concentration (Eq. 1). Then the total amount of calcium released (Ca_total_) and the calcium release flux (FL) through the RyRs was calculated (Eq. 2). It is clearly visible in Fig. 5, that the change in intracellular calcium concentration (panel B and D) as well as the total calcium released into the myoplasm (panel E), and the corresponding calcium release flux (panel F) was increased significantly in selenate treated mice. The amount of released calcium showed an about 60 and 100 % increase in 0.5 ppm NanoSe and selenate fed mice, respectively. The data presented in Fig. 5 clearly demonstrate that depolarization-evoked calcium release from the SR was significantly higher in selenium fed mice.

#### Western blot analysis of selenoprotein expression

**Fig. 6:**
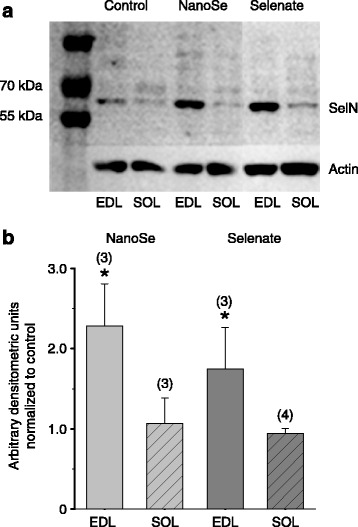
Selenoprotein N expression increased in selenium treated mice. Representative Western blot image showing the expression of selenoprotein N in EDL and SOL muscles from a control mouse and a mouse fed with 0.5 ppm NanoSe or selenate for two weeks (**a**). Actin was used as loading control. Quantified expression of selenoprotein N in EDL and SOL muscles of selenium treated mice normalized to actin and control (**b**). * denotes significant difference from control at level *P* < 0.05. Numbers in parenthesis give the number of animals fed with 0.5 ppm NanoSe or selenate

Finally the expression level of selenoprotein N in EDL and SOL muscles from control mice and mice fed with NanoSe or selenate was determined. In 0.5 ppm concentration both selenium compounds increased significantly the SelN expression only in EDL muscle (Fig. 6a and b). However, in 5 ppm concentration both selenium compounds increased SelN expression significantly in both (EDL and SOL) muscle samples (data not shown).

## Discussion

The results of our study provide novel insights into the effects of dietary selenium on skeletal muscle functions. New findings of the study are the direct effects of selenium supplementation on skeletal muscle function.

### Selenoprotein in selenium fed animals

One explanation of the increased force we observed on the selenium fed mice could be the alteration of the expression of selenoprotein N. Arbogast and Ferreiro [16] have shown that SelN deficiency is associated with abnormalities in intracellular Ca^2+^ handling, potentially related with RyR1 dysfunction in myotubes. Another study on rats presented that the lack of selenoprotein causes diaphragm muscle weakness by lowering diaphragmatic glutathione peroxidase activity, and then these animals are subjected to the oxidative stress of resistive loading [28].

We examined the selenoprotein N expression of muscles and found it markedly increased in EDL (Fig. 6). This is in good agreement with previous studies [29, 30], where selenium supplementation caused a similar increase in the level of several selenoproteins. In our experiments a low dose of selenium produced favorable effects on skeletal muscle functions, presumably by increasing the intracellular availability of selenium for selenoprotein N synthesis and stimulating SelN expression.

### Toxicity and efficacy

Several animal studies showed long time ago that Se deficiency may result in damage to the heart, liver, kidneys, lung and skeletal muscle [7, 29, 31, 32]. On the contrary, only recent investigations aimed to explore the effects of increased Se supplementation [13, 14, 29, 30], but none of them studied skeletal muscle functions. The doses used in our study (0.5 and 5 mg/kg) were similar to doses used in a previous study [13] in which both were shown not to be toxic in mice. Though in this study both selenium compounds decreased the white blood cell number and the weight of spleen in the higher concentration, only selenate decreased the weight of liver. These changes were significant but did not lead to the death of animals. We point out that all animals survived the 2-week-long treatment in our experiments and a minor increase in body weight was observed proving the sub toxic dose of selenium. On the other hand, the higher Se dose (5 mg/kg) caused significant decrease in body weight of rats, but not in mice after 14 days [33]. However, a 12 weeks application of 0.5 mg/kg sodium selenite induced reactive myocardial fibrosis and systolic dysfunction in mice [29]. This suggest that there is a narrow range between the effective and toxic concentration of selenium and its accumulation needs also to be considered in further therapy.

We did not find obvious difference in the efficacy of the two selenium compounds. In *in vivo* experiments they were equally effective. NanoSe increased more the force in EDL than selenate, however their efficacy was the same in soleus. This can be explained considering the fact that NanoSe increased the SelN expression in EDL more than selenate, whereas this effect was equal in the slow muscle. Surprisingly selenate increased more the amplitude of calcium release from the SR. Similar bipolar effects were observed in a recent study [34] where the efficacy and toxicity of selenium nanoparticles and selenium dioxide were compared. Whereas the former showed higher scavenging activity and lower cytotoxicity in cell culture, the latter had higher electron-donating activity.

### Regulation of EC-coupling

It has been known for a long time that muscular activity increases the production of reactive oxygen species, and this seems to correlate with the loss of muscle function [35–37]. Exogenous antioxidants given prior to the onset of muscle activity, delay or minimize the decline in muscle performance and decrease the effects of the oxidative stress [37, 38]. However, the relationship between the function of the different cellular antioxidant systems and the performance of skeletal muscles remains unclear. It was shown recently that reactive oxygen species is a physiological regulator of skeletal RyR [39]. Oxidation of RyR resulted in increased RyR activity and Ca^2+^ leak from the sarcoplasmic reticulum in rest. This leads to reduced SR calcium content and finally decreased force production. The antioxidant effect of selenium can reverse this and result in a higher force as observed here. Similar effects were found in a study on diabetic rats demonstrating a cardio protective action of selenium and suggesting that one of the underlying mechanisms is the protection of cardiac ryanodine receptor Ca^2+^ release channel against oxidative stress [40].

SelN can also regulate the calcium level in the SR via enhancing SERCA activity by reducing the luminal H_2_O_2_-oxidized cysteines [41]. This is clearly visible in Fig. 5b showing the faster declining phase (i.e. higher pumping rate of SERCA) after depolarization in case of both selenium compounds. In the absence of SelN, excessive oxidation of SERCA results in reduced reuptake of calcium and an increase of cytosolic calcium at rest. This again results in a decrease in SR calcium content and associates with decreased muscle force.

It was also shown in zebrafish embryos that both SelN and RyR are required for normal muscle development. These two proteins are physically associated *in vivo* and SelN is necessary for full activation of RyR [42]. Our observation that Se supplementation increased the SelN level may also explain the increased intracellular calcium concentration change during depolarization. Combining its antioxidant effects on EC-coupling components with the latter the result could be an improved force production of skeletal muscle.

### The effects of selenium on muscle performance

Several studies investigated the effects of selenium supplementation on body weights in chickens [43], lambs [44], cows [45], and also in laboratory animals like rats [46] and mice [13]. The results are diverse depending on the concentration, duration, and source of selenium. We found a slight but significant body weight and relative muscle weight increase in our experiments. This is in good agreement with a recent study in which fish oil and selenium supplementation was able to increase body and muscle weight in pathological conditions [47]. The authors gave a plausible explanation that the supplementation of selenium and fish oil improved the skeletal muscle atrophy due to down-regulation of myostatin and related cytokines. This pathway can work also in control condition and it is known that the inhibition of myostatin leads to extraordinary increase in muscle weight [20].

Even though selenium is widely used as dietary supplement alone or in combination with vitamins, its effects on muscle performance is under-investigated. Up to now only one clinical trial was conducted on humans [48]. In this study vitamin E, vitamin C, zinc, and selenium supplementation for 17 weeks improved maximal voluntary contraction, and endurance limit time of quadriceps by reducing oxidative stress and enhancing the antioxidant defenses. We found similar positive effects on force in EDL and SOL of mice just after 2 weeks application of selenium alone. Although, the whole muscle experiments were done on EDL and SOL, while isolated muscle fiber experiments were on FDB the results are reconcilable, since EDL and FDB contains essentially only fast-twitch fibers. The higher intracellular [Ca^2+^] change during depolarization in FDB fibers from Se supplemented mice is well correlated with the higher force development in EDL from the same animal. The fact that the human study was conducted on Facioscapulohumeral muscular dystrophy patients, underline the potential application of selenium in medical treatment.

## Conclusion

In conclusion, selenium has positive effects on the muscle performance of mice and NanoSe takes its effects already in low concentration. It increases the releasable calcium in the intracellular stores and the selenoprotein N expression in the muscles. Our results suggest that an antioxidant strategy with a low dose selenium supplementation could be a relevant therapeutic approach for patients with myopathies and could also serve as counteracting sarcopenia in the elderly.

## Additional file

Additional file 1: Figure S1. Photo of bacterias (B) and elemental selenium nanoparticles (globular objects with 200-500 nm diameter) by scanning electron microscope. The elemental selenium nano-sized balls (NanoSe) have red color in truth. Figure S2 150 tetani were elicited at 0.5 Hz during 5 min. The amplitude of tetani was normalized to the first tetanus. (PDF 280 kb)

## Abbreviations

[Ca^2+^]_i_: Intracellular calcium concentration
A/D: Analog to digital
AM: Acetoxymethyl
ANOVA: Analysis of variance
BCA: Bicinchoninic acid
BSA: Bovine serum albumin
Ca_total_: The total Ca^2+^ in the myoplasm
Ca_transp_: The amount of Ca^2+^ transported by the Ca^2+^ pumps
DHPR: Dihydropyridine receptor
DNA: Deoxyribonucleic acid
DTT: Dithiothreitol
EC-coupling: Excitation contraction coupling
EDL: *extensor digitorum longus* muscle
EGTA: Ethylene glycol-bis (β-aminoethyl ether)-N,N,N',N'-tetraacetic acid
F_340_, F_380_: Fluorescence intensity at 340 or 380 nm illumination
FDB: *flexor digitorum brevis* muscle
FL: Flux
GPx: Glutathione peroxidase
GSH: Reduced glutathione
HRP: Horseradish peroxidase
Kd: Dissociation constant
MRS: Liquid culture medium for the isolation of lactobacilli, according to de Man, Rogosa and Sharpe
NanoSe: Nano-sized (100 to 500 nm diameter) elemental selenium
PBS: Phosphate-buffered saline
PBST: PBS supplemented with 0.1 % Tween 20
ppm: Particle per million
RDA: Recommended daily allowance
RyR: Ryanodine receptor
SelN: Selenoprotein N
SEM: Standard error of the mean
SEPN1: Gene encodes selenoprotein N
SERCA: Sarcoplasmic reticulum calcium pump
SOL: *musculus soleus*
SR: Sarcoplasmic reticulum
T-tubule: Transverse tubule
