# Supplementary material for: Dietary selenium augments sarcoplasmic calcium release and mechanical performance in mice
Source: Nutr Metab (Lond). 2016 Nov 3;13:76. doi: 10.1186/s12986-016-0134-6 (PMC5094064; doi:10.1186/s12986-016-0134-6)
Supplement: Additional file 1: Figure S1. — Photo of bacterias (B) and elemental selenium nanoparticles (globular objects with 200-500 nm diameter) by scanning electron microscope. The elemental selenium nano-sized balls (NanoSe) have red color in truth. Figure S2 150 tetani were elicited at 0.5 Hz during 5 min. The amplitude of tetani was normalized to the first tetanus. (PDF 280 kb) [file 12986_2016_134_MOESM1_ESM.pdf]

Supplementary figure 1

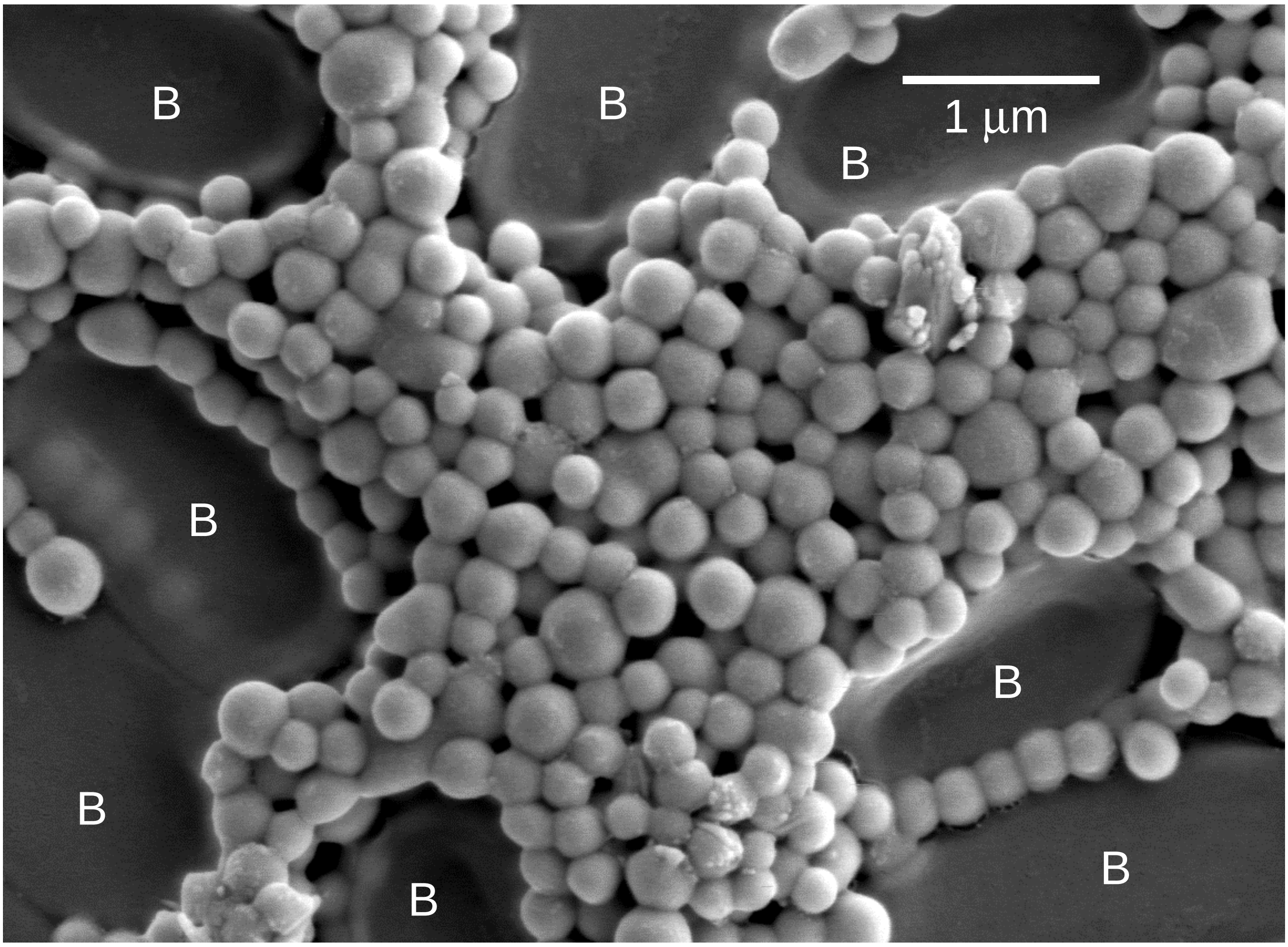


Photo of bacterias (B) and elemental selenium nanoparticles (globular objects with 200-500 nm diameter) by scanning electron microscope. The elemental selenium nano-sized balls (NanoSe) have red color in truth.

# Supplementary figure 2

## Fatigue

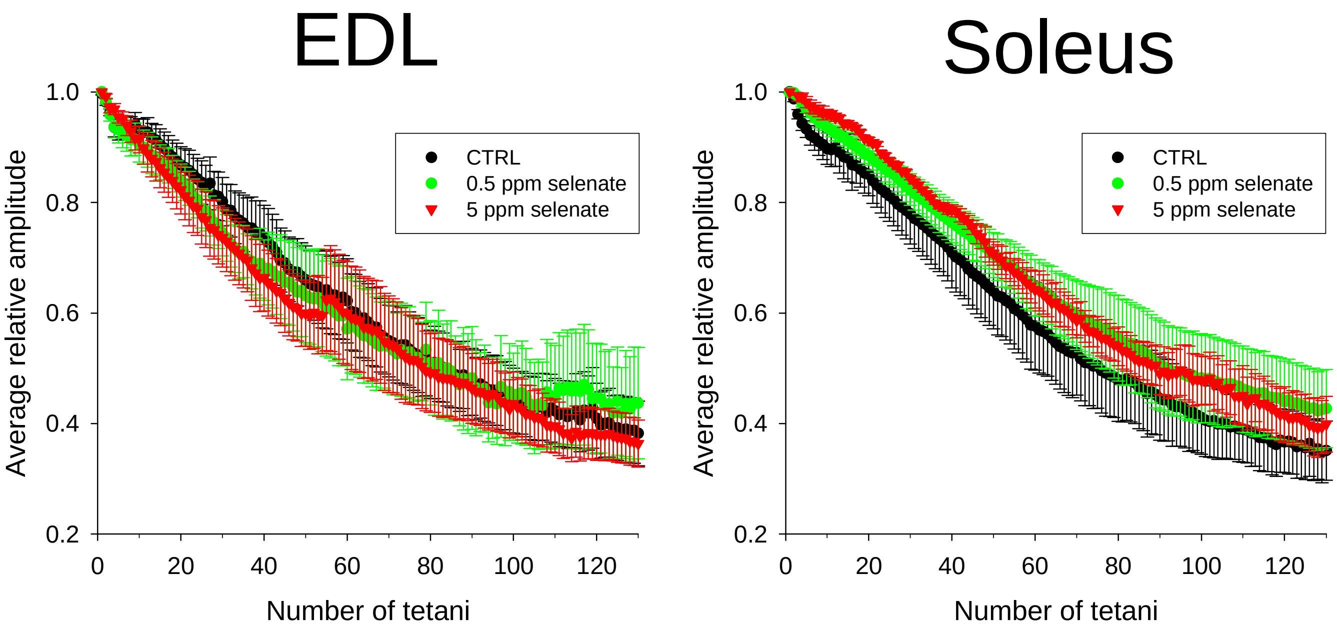


150 tetani were elicited at 0.5 Hz during 5 minutes. The amplitude of tetani was normalized to the first tetanus.

NanoSe has same effects on fatigue.
